# Nurse Practitioners and Men’s Primary Health Care

**DOI:** 10.1177/1557988315617721

**Published:** 2015-11-26

**Authors:** Marina B. Rosu, John L. Oliffe, Mary T. Kelly

**Affiliations:** 1 University of British Columbia, Vancouver, British Columbia, Canada

**Keywords:** men’s health, primary health care, nurse practitioner, health promotion, illness prevention, masculinity

## Abstract

Though life expectancy sex differences are decreasing in many Western countries, men experience higher mortality rates at all ages. Men are often reluctant to seek medical care because health help-seeking is strongly linked to femininity, male weakness, and vulnerability. Many men are also more likely to access emergency care services in response to injury and/or severe pain instead of engaging primary health care (PHC) services. Nurse practitioners are well positioned to increase men’s engagement with PHC to waylay the pressure on emergency services and advance the well-being of men. This article demonstrates how nurse practitioners can work with men in PHC settings to optimize men’s self-health and illness prevention and management. Four recommendations are discussed: (1) leveling the hierarchies, (2) talking it through, (3) seeing diversity within patterns, and (4) augmenting face-to-face PHC services. In terms of *leveling the hierarchies* nurse practitioners can engage men in effectual health decision making. Within the interactions detailed in the *talking it through* section are strategies for connecting with male patients and mapping their progress. In terms of *seeing diversity* with*in patterns* and drawing on the plurality of masculinities, nurse practitioners are encouraged to adapt a variety of age sensitive assessment tools to better intervene and guide men’s self-health efforts. Examples of community and web based men’s health resources are shared in the *augmenting face-to-face PHC services* section to guide the work of nurse practitioners. Overall, the information and recommendations shared in this article can proactively direct the efforts of nurse practitioners working with men.

## Introduction

There is disparity in mortality between men and women; in the United States, Canada, and many other developed countries men’s life expectancy is 4 to 6 years shorter than that of women (World Health Organization, 2012). Men also access fewer preventive and primary health care services than women (Nabalamba & Millar, 2007; Pinkhasov et al., 2010). For example, amid many men’s reticence for seeking medical screening tests, services often fail to engage and/or orientate men to primary health care (PHC) services and providers. In addition, studies report that men often times access emergency departments for conditions that could be addressed in outpatient settings (Canadian Institute for Health Information [CIHI], 2012a), a confounding situation for addressing men’s health. The purpose of this article is to describe theory based and practical issues related to men’s health and make recommendations for how the role of nurse practitioners can help increase men’s uptake of PHC services and waylay the pressure on emergency services to advance the well-being of men and their families.

## Defining Primary Health Care

Within this article, PHC refers to medical services offered to patients at medical offices, walk-in clinics, community health centers or family health teams and administered by health care providers including physicians and nurse practitioners. PHC combines medical care with other health services that encompass health promotion and primary prevention (Romanow, 2002). The hallmark of this approach is interdisciplinary health care provider collaboration to increase patient accessibility to resources at the community level. The benefits include improved quality of care and efficient use of finite resources. Canada has been striving toward integrating nurse practitioners as primary care providers, but despite benefits of the role, significant challenges remain (Donald et al., 2010). In the United States, nurse practitioners are viewed as extremely important to the future of high quality PHC, though variation in state laws, disparate payment policies, and interdisciplinary tensions have posed barriers to their full integration (Naylor & Kurtzman, 2010). Nonetheless, PHC teams that include nurse practitioners can improve patient follow-up and disease management by advancing effectual health promotion and disease prevention for men.

## Episodic Medical Care and Men’s Health

Despite a move toward interdisciplinary PHC teams, health care in Canada and the United States is often taken up on an episodic basis. For example, men tend to visit PHC providers for acute problems and then desist when the problem ceases. Within fee-for-service models, physicians also usually limit consultation times, triaging treatment above illness prevention counselling. As such, prominent risky male behaviors including tobacco, alcohol and marijuana overuse, and poor dietary practices (Pinkhasov et al., 2010) can continue despite receiving PHC for a diagnosed disease. To counter limited consult times and a focus on treating disease, physicians may educate patients during annual or biannual comprehensive physical exams when they are able to book longer consultations (MacMillan & Goldenberg, 2011).

There are several challenges to the aforementioned approaches, particularly in regard to men’s health and their uptake of PHC. For example, in 2007, the American Academy of Family Physicians (AAFP) conducted a men’s health study to evaluate the frequency with which men attended PHC, their willingness to do so, and the contexts of those visits. Among the 756 men, 18 to 54 years old, 48% to 66% had had a physical exam within the previous 2 years; among the 401 men 55 years and older, 84% had had a physical exam within that time period (AAFP, 2007); however, 36% of surveyed men had visited their health provider only when extremely sick, and 29% had waited as long as possible before seeking medical care. Mansfield, Addis, and Courtenay (2005) suggest many men are unlikely to visit a PHC provider or to have a regular physician. Similar trends exist among Canadian men. A survey of more than 120,000 Canadian respondents concluded that women 18 to 64 years of age were far more likely than men to have a GP consult, multiple GP consults or a specialist consult. Similarly, a study that monitored men’s and women’s health care utilization for 1 year concluded that men showed lower patterns of utilization and lower associated costs (Bertakis, Azari, Helms, Callahan, & Robbins, 2000). These sex differences held when adjustments were made for the effects of chronic conditions and pregnancy and birth-related consults were excluded (Nabalamba & Millar, 2007). Similarly, a study using a U.K.-wide primary care database reported that, overall, men were 30% less likely to consult a primary care physician (Wang, Hunt, Nazareth, Freemantle, & Petersen, 2013). The authors reported that this trend was most robust for middle-age males, and these sex differences diminished among older adults. The differences were also negligible in comparisons of men and women who were already receiving medication (Wang et al., 2013).

Other researchers argue that young men from low socioeconomic backgrounds who often times have sedentary lifestyles and poor diets are the men most estranged from PHC (MacMillan & Goldenberg, 2011). This subgroup is also less likely to be informed about diet and exercise—or resources to make healthy choices, which in turn renders them more susceptible to premature cardiovascular disease and other chronic illnesses (Honda, 2004). The Young Men’s Health Consultation Report (Planned Parenthood of Toronto [PPT], 2005) shared findings drawn from focus group interviews to describe the underlying issues, gaps and barriers for accessing PHC among Toronto-based men 16 to 25 years old (*n* = 41). The results indicated that participants experienced negative interactions with health care professionals, long wait times, and misdiagnoses in conceding that a lack of confidence in their providers underpinned widespread reticence to further engage PHC (PPT, 2005). The men also asserted that they had no health issues and therefore did not “need” to regularly consult a doctor for screening or check-ups (PPT, 2005). While young men may be unlikely to access PHC, older, educated males with higher health literacy levels are more frequent users (MacMillan & Goldenberg, 2011).

It is important to note that the evidence on sex differences, gender, and health care utilization is complex and somewhat inconsistent. Studies focused on specific patient populations or symptoms have reported negligible differences between men and women’s use of general practitioners. For example, Wyke, Hunt, and Ford (1998) examined the pattern of women and men’s consults for minor illnesses and concluded their data did not support the widely held claim that women are more likely than men to consult for an illness symptom. Similarly, a study that investigated the pattern of GP consults among cancer patients before their diagnoses found little difference between males and females in the 24 months prior to their diagnosis (Wang, Freemantle, Nazareth, & Hunt, 2014). A study that compared male and female consult patterns for headache and backache also concluded weak evidence exists for sex-based help-seeking differences in regard to these specific symptoms (Hunt, Adamson, Hewitt, & Nazareth, 2011). These studies did not however detail the content, length or quality of patient consults, the communication styles, nor the influence of masculinities and femininities among their samples. Qualitative research with cardiac patients (men and women) who delayed seeking treatment has underlined the importance of a nuanced approach to sex and gender by incorporating and mapping plural understandings of masculinity and femininity (Galdas, Johnson, Percy, & Ratner, 2010).

From a broader perspective, Jasek (2011) identified that having a regular PHC provider garnered preventative services regardless of insurance status and ethnicity—yet those connections can be especially challenging to make with male patients. For example, in Canada males 20 to 64 years of age are far more likely than females to *not* have a regular doctor (Statistics Canada, 2015). On average almost 20% of Canadian men, compared with 10% of women, report not having a regular doctor. Among young men 20 to 34 years old, more than 30% report not having a doctor compared with 20% of women in this age category. On the occasions that men do visit a PHC provider, their presenting problems tend to fall into specific categories: sexuality issues (e.g., low libido), chronic illness (e.g., diabetes), and injuries related to sports or work (e.g., fractures; MacMillan & Goldenberg, 2011).

Outside episodic care, annual/biannual screening can connect patients with PHC on a regular basis. Many women, for example, complete annual/biannual Papanicolau and mammography exams (Poole, Gelmon, Borugian, Kan, & Stilwell, 2008). In contrast, the Canadian Urological Association discourages annual prostate examinations and prostate specific antigen monitoring in patients who are low risk (Izawa et al., 2011). The lack of routine male-specific screenings can be reasonably argued as missed opportunities for education and illness prevention. MacMillan and Goldenberg (2011) confirm that screening is not enforced due to a lack of available resources and materials targeting men, suggesting PHC systems also fail to capture men’s attention for screening on the occasions when men do access services. Men who are middle-aged and older may benefit by early detection and preventative services such as colonoscopy, cholesterol and blood pressure screenings, pneumococcal, and seasonal influenza vaccinations. Lack of preventative care can result in untreated advanced disease due to emergent illnesses and/or missed opportunities for curative treatment options (Addis & Mahalik, 2003). As such, heart disease, colon cancer, pneumonia, and influenza are common causes of men’s mortality (Heron et al., 2009).

In summary, episodic care in men’s health is driven by system and social issues, which can merge to negatively influence men’s health practices and illness experiences. PHC services, and the uptake of those resources by men, are deeply reliant on the benefits and experiences derived by the men engaging those services. In this regard, nurse practitioners in particular might thoughtfully consider the interconnections between men, masculinities and PHC to maximize the positive impact of their emergent role.

## Men, Masculinities, and PHC

Masculinities refer to men’s socially constructed and culturally idealized practices (Connell, 2005). Masculine norms in the form of competitiveness, strength, power, and self-reliance contribute to the ways in which men do and do not do self-health (Oliffe & Phillips, 2008). Avoidance of PHC services can flow from men’s desire to be autonomous and self-reliant (Galdas, 2013). Some men’s disregard for health promotion, especially those embedded in discourses around injury and illness prevention, are also tied to gender ideals that position caring for one’s health as synonymous with feminine identities (Courtenay, 2000). By contrast, health risk taking activities seen as masculine include smoking and alcohol overuse, both of which contribute significantly to men’s morbidity and mortality rates (Starfield, Shi, & Macinko, 2005). Early detection or surveillance of an emergent condition can also create uncertainty and erode men’s claims on masculine ideals including strength and control (Galdas, Cheater, & Marshall, 2005). Accessing health care for specific issues—most notably pain—is acceptable, and congruent with masculine codes of behavior such as stoicism, especially when that pain has increased with time and/or prevailed despite self-management attempts.

Contrasting the connections between masculinities and men’s poor health outcomes, work by Sloan, Gough, and Conner (2010) and Oliffe, Ogrodniczuk, Bottorff, Johnson, and Hoyak (2012) has described how some masculine ideals are operationalized to positively advance men’s health outcomes. For example, Oliffe et al. (2012) detailed how ascribing to protector and provider roles afforded men experiencing depression and suicidal thoughts protection from self-harm. Further confirming the transformative aspects of masculinities, Creighton and Oliffe (2010) described how men’s masculinities are contextual and relational, emerging diversely within various communities of practice, often times to benefit men’s health. Integrating this evidence, it is key to acknowledge that while men have historically been somewhat disconnected from PHC, change is imminent, and great potential resides for nurse practitioners to engage men with PHC services.

## Emergency Care and Men’s Health

Emergency departments (EDs) are treatment facilities designed for acute, episodic care of patients experiencing acute injuries, illness, and/or exacerbation of chronic disease (Moineddin, Meaney, Agha, Zargorski, & Glazier, 2011). Fifty to 57% of men treated in the ED present with nonurgent conditions (CIHI, 2005; McCaig & Burt, 2005). Some men visit EDs because they do not have a PHC provider, whereas others access EDs because they are open 24/7 and have specialty services and diagnostics (Canadian Association of Emergency Physicians and National Emergency Nurses Affiliation, 2001). As a result of patient demand and finite ED resources, long wait times, crowding and ambulance diversions often ensue (Moineddin et al., 2011). Almost 60% of the more than 1,100 American men surveyed said that “something prevents them from going to the doctor” and more than one-third of American men aged 18 to 54 said they wait until they are extremely sick before seeking medical help (AAFP, 2007).

Ambulatory care sensitive conditions (ACSCs) including asthma, chronic obstructive pulmonary disease (COPD), diabetes, hypertension, and other cardiovascular diseases, do not usually require hospital admissions if men access appropriate PHC services early on (CIHI, 2012a; Northern Health, 2013). A 2008 survey of PHC users (*n* = 4,138) reported that 22% (*n* = 910) of men diagnosed with ACSC had had no contact with a PHC provider in the past 12 months, while 11% (*n* = 455) of men reported that their last visit to the ED was for a condition that they perceived as being treatable by a PHC provider (CIHI, 2012a). What follows are the top complaints that men present with to the ED: (1) respiratory, (2) trauma, (3) cardiovascular, and (4) mental illness, substance overuse, and liver toxicity (CIHI, 2005).

### Respiratory

Of the men treated for asthma exacerbation in the ED, 70% to 90% are discharged home (Lazarus, 2010). In a study of 100 patients, the strongest predictors of repeat visits to the ED were being male and having a previous history of ED visits (Pai et al., 2014). Within this study, nearly half of participants reported not using maintenance inhalers, likely because these inner city patients had limited access to PHC, could not afford the medications, and/or preferred to avoid long-term use of medications (Pai et al., 2014). Patients who visit EDs for acute exacerbation of COPD are also more likely to be male, older (55-74 years) and of low socioeconomic status (Hasegawa, Tsugawa, Tsai, Brown, & Camargo, 2014). ED visits for COPD can be prevented through PHC interventions including smoking cessation programs, influenza and pneumococcal vaccines, educating around the correct use of inhalers, and evidence-based pharmacology treatment (Hasegawa et al., 2014).

### Trauma

In younger male populations (<20 years old), trauma is a common reason for ED visits. Seventy-five percent of the head injuries seen in EDs are men, and burns (Canner et al., 2014), motor vehicle accidents, assaults, and firearm injuries also feature among men presenting at the ED (Allareddy et al., 2014). These statistics reveal men as a target group for prevention programs, and within the context of PHC, effectual men-centered programs including a focus on safety issues such as helmet and seat belt use could quell the male disabilities and deaths attributed to these preventable traumas (Koestner, 2012).

### Cardiovascular

The prevalence of cardiovascular disease (CVD) is high among men, especially in men 55 to 64 years old, resulting in high rates of CVD-related mortality (Goldenberg, 2012). In 2009, 49% of male deaths were attributed to heart disease (Go et al., 2013). Palpitations are a common reason why men attend the ED (Probst et al., 2014) and a third of these visits are linked to an acute cardiac event (Probst et al., 2014). Chest pain, hypertension, and heart failure are other common CVD-related reasons for male ED visits (Czarnecki et al., 2014; Kleinschmidt et al., 2014; Storrow, Jenkins, & Self, 2014). CVD risk factors such as smoking, poor diet and sedentary lifestyles are modifiable and therefore, preventable, especially within PHC (Farrell & Keeping-Burke, 2014; Harvard University, 2014).

### Mental Illness, Alcohol Overuse, and Liver Toxicity

Men are frequently assessed in the ED for mental illness, alcohol intoxication and/or opioid overdose and liver toxicity (Chakravarthy et al., 2013; Hasegawa, Brown, Tsugawa, & Camargo, 2014; Verelst, Moonen, Desruelles, & Gillet, 2012). Usually these issues are interrelated and challenging to treat holistically within noisy, hectic ED environments that typically specialize in treating physical ailments (Chakravarthy et al., 2013). Downstream, suicide is four times more common in men compared with women and risk factors include depression, social isolation and/or alcohol overuse (Centers for Disease Control and Prevention, 2015). Men do not routinely seek professional help for depression (Johnson, Oliffe, Kelly, Galdas, & Ogrodniczuk, 2012), and it can be challenging for men to articulate their mental health concerns to PHC providers (Wide, Mok, McKenna, & Ogrodniczuk, 2011). Instead, men often times express depression as anger and irritability, and are likely to self-medicate with alcohol and other drugs (Oliffe & Phillips, 2008). Traditional treatment for depression relies on pharmacotherapy and/or counselling (Busch & Sandberg, 2012) and an effective alternative counselling method for men can be all-male groups that offer a safe environment (Ogrodniczuk & Oliffe, 2009). In terms of PHC services, men could derive great benefits from mental health promotion efforts and treatment regimens to avoid or reduce the frequency of acute episodes of mental illness.

## Nurse Practitioners and Male Patients in PHC

Nurse practitioners in PHC often focus on health promotion and disease prevention (Worster, Sarco, Thrasher, Fernandes, & Chemeris, 2005). By optimizing nurse practitioners’ approaches to men’s health, men’s uptake of PHC can be increased and ED visits reduced while advancing the well-being of men and their families. Of course, the challenge is how to engage men in PHC. Four recommendations follow toward achieving the aforementioned interconnected goals.

### Leveling the Hierarchies

Historically, many men have been somewhat passive in their interactions with health care providers due to the power differentials that are implicit (and sometimes explicit) to patient’s vulnerabilities, and the provider’s expert status, education, and knowledge base (Alexander, Hearld, Mittler, & Harvey, 2012). Visible minority men, in particular, report significant barriers and challenges in obtaining screening and health information (James, Salganicoff, Ranji, Goodwin, & Duckett, 2012). For example, many African American men do not use preventative health services due to socioeconomic barriers and/or lack of trust (Hammond, Matthews, & Corbie-Smith, 2010). Hispanic men can also mistrust health care providers, and fear being “guinea pigs” and/or embarrassed within the context of asking for professional help (Davis, Bynum, Katz, Buchanan, & Green, 2012). Central to this situation is the concept that masculine ideals shape men’s social relationships, often emphasizing as well as being sensitive to power and dominance (Connell, 2005). In health settings, a hierarchical, paternalistic relationship with care providers can deter and disenfranchise some male patients, quelling their interest and energy for accessing health promotion programs (Watson, 2000).

Patient–provider relationships that include humor, empathy, and frankness can overcome power differentials between men and HCPs (Smith, Braunack-Mayer, Wittert, & Warin, 2008a). Men’s involvement in their health care can be optimized when they understand their role and are affirmed in their engagement, knowledge, and confidence to have and express opinions and preferences (Hibbard, Stockard, Mahoney, & Tusler, 2004). Within this context, nurse practitioners can promote therapeutic health care interactions characterized by trust (Lubetkin, Lu, & Gold, 2010) and equitable partnerships (Hibbard & Cunningham, 2008). Nonpharmacological interventions are a good place to start, because such behavioral and lifestyle approaches give men charge over their health, alleviating feelings of vulnerability or loss of control (Harris & McKenzie, 2006). When designing health interventions nurse practitioners can also apply a shared decision-making model whereby male patients review the available options, weighing up benefits/consequences in consultation to reach *his* best course of action (Bendix, 2014).

The vast majority of nurse practitioners are female (93%; CIHI, 2012b), and female HCPs have been noted to engage in more active partnership behaviors, for example, more open, emotionally focused, and patient-centered talk with their clients (Roter, Hall, & Aoki, 2002). Although men tend to favor a concise, direct and to-the-point style of communication based on a trusting relationship with their HCP (Smith, Braunack-Mayer, Wittert, & Warin, 2008b), many male patients also have a greater level of comfort, engagement, disclosure, and assertiveness when speaking to female health providers (Hall & Roter, 2002). This may be due to the more open communication style and the perception of reduced power differentials related to gender, as well as other more complex reasons within the context of the consult. Forging trust, rapport, and reducing power dynamics can help men engage more readily with nurse practitioners (Alexander et al., 2012).

### Talking It Through

Motivational interviewing is a counselling style that can resolve men’s ambivalence for self-health by challenging their thinking and expectations toward a readiness to change (Rollnick & Miller, 1995). Evoking and facilitating the release of a man’s inner motivation and resources for change, motivational interviews can be a valuable tool for nurse practitioners (Rollnick & Miller, 1995). For example, positive results have been leveraged though motivational interviewing to change men’s alcohol and dietary behaviors (B. L. Burke, Arkowitz, & Menchola, 2003). Prochaska and Velicer’s (1997) transtheoretical model of behavior is also a useful tool for individualizing and mapping care based on the six stages of change. It might also be useful for nurse practitioners to engage men’s partners in supporting them with health-promoting behavior changes (V. Burke, Giangiulio, & Gillam, 2004) because significant others, especially female spouses, can be influential in men’s sustained positive self-health and help-seeking behaviors (Forbat, Place, Hubbard, Leung, & Kelly, 2014). Many nurse practitioner programs teach these techniques, and motivational interviewing (Rongkavilit et al., 2015) has revealed significant benefits for male clients as have transtheoretical approaches (Brooks, 2010) to providing and mapping men’s care.

Central to these and other techniques is the need to explicitly provide men with the permission to talk, and ask open-ended questions along with the use of prompts and probes to solicit contextual information (Oliffe & Mroz, 2005). Mapping men’s progress via the transtheoretical model or similar is also key to gauging progress and strategizing efforts to garner men’s talk. These approaches trade on masculine ideals of autonomy and self-reliance by working directly with men to promote self-health and illness management. Inversely, stereotypical expectations that men tend not to talk and/or the use of closed ended questions invariably yield *yes*-*no* answers from most men.

### Seeing Diversity Within Patterns

How men respond to PHC depends on an array of factors and experiences including their social relationships, age, disability, income levels, ethnicity, sexual orientation, socioeconomic status, and education (Robertson, 2006). In turn such factors affect how men learn, or change their behaviors (Robertson, 2006). For example, men can interpret changing their behavior(s) as surrendering part of their manliness (Connell & Messerschmidt, 2005). With this knowledge, nurse practitioners should try to operationalize men’s behavior changes as strength based (Oliffe, Bottorff, & Sarbit, 2012). Therefore, the language used in lobbying self-health should focus on men’s decisiveness, resilience, and autonomy—all of which play to many men’s masculine ideals (Oliffe, Bottorff, et al., 2012). Nurse practitioners can also garner sustainable behavior changes by focusing on small incremental changes and then identifying specific men-friendly health promotion strategies to support those efforts (Rollnick, Mason, & Butler, 2000).

By recognizing that masculinities—and therefore men as plural, diverse, and changing over time (Connell, 2005)—nurse practitioners can tailor their engagement with men according to individual needs and specific contexts and time points (Williams & Robertson, 2006). It is important for nurse practitioners to avoid assuming and enforcing traditional male roles or stereotypes in their interactions with male patients, or limiting care to physical assessments and “lifestyle” advice because such reductionist approaches neglect many related men’s health issues (Williams & Robertson, 2006). Instead, it is critical to assess the man’s alignment to masculine ideals as a means to building relationships to empower them to develop skills and strategies relevant and effectual in advancing their health (Williams & Robertson, 2006). Related to this, nurse practitioners should use a variety of comprehensive assessment tools to work with male patients. For example, Goldenring and Rosen (2004) recommended the HEADSS tool for assessing adolescent males in PHC. The HEADSS tool is a mnemonic that prompts nurse practitioners to ask questions about the *H*ome environment, *E*ducation and employment, social *A*ctivities, *D*epression and suicide risk, *S*afety from injury and ***S***exuality, including erectile dysfunction. Major causes of morbidity and mortality among adolescents include mental illness, concerns about sexual health, and relationships with significant others (Goldenring & Rosen, 2004). Adolescent males are unlikely to articulate these concerns, and instead may present with minor complaints, such as a headache, which can mask serious problems including depression, anxiety, an eating disorder, drug overuse, problems at school, and/or an interpersonal conflict(s). The HEADSS health assessment can also help nurse practitioners develop rapport with male youth while also identifying areas for intervention and health promotion (Goldenring & Rosen, 2004).

Men over the age of 40 have high prevalence of ischemic heart disease leading to significant mortality and morbidity (Harris & McKenzie, 2006). Their increased cardiovascular risk can reflect smoking, alcohol overuse, poor diets, and obesity (Harris & McKenzie, 2006). These are all modifiable risk factors that nurse practitioners should anticipate among men older than 40 and offer appropriate interventions and counselling (Berra, 2010). The Royal Australian College of General Practitioners (2015) proposed the SNAP tool—*S*moking, *N*utrition and weight, *A*lcohol and *P*hysical activity—to evaluate risk factors that predispose men to CVD. Health care providers also use the 5As (ask, assess, advise, assist, arrange) approach in conjunction with SNAP to plan and provide preventative care (Royal Australian College of General Practitioners, 2015). This approach can be used in combination with the aforementioned transtheoretical model of change and motivational interview techniques. Among men older than 50 years and nearing retirement, Tan et al. (2014) propose the men’s health index, a promising tool comprising 10 instruments, specific to men’s health to assess frailty and fitness and to predict life expectancy, morbidity, and mortality. Each category includes strategies for improving health and function. The men’s health index is also used to assess mental health, smoking, erectile dysfunction, lower urinary tract  symptoms, and testosterone deficiency (Tan et al., 2014).

In “seeing diversity within patterns” nurse practitioners should use age specific assessment tools to better formulate and familiarize themselves with the health needs of men at diverse life stages. Similarly, recognizing diversity within and across men is key to nurse practitioners’ thoughtful and thorough assessments of males.

### Augmenting Face-to-Face PHC Services

During a PHC visit, men are likely to have shorter consultations compared with females (Britt, Valenti, & Miller, 2005), which translates into reduced illness prevention education. It is important to provide multiple opportunities and diverse mechanisms to engage men (Strange & Tenni, 2012), for instance, by making a follow-up appointment to participate in an illness prevention conversation, and/or by recommending community or web-based resources (Robertson & Williamson, 2005). Nurse practitioners are excellent resources for pointing men toward community-based interventions. For example, Australia’s Men’s Health Model of Practice at Bendigo Community Health Services connects community health promotion with a men’s health clinic, provided by a nurse practitioner (Strange & Tenni, 2012). Initial clinic appointments are long enough to allow a comprehensive health assessment after which the nurse practitioner provides patient education and opportunities for action. Occasionally the nurse practitioner also provides services at patients’ workplaces and/or offers after-hours consultations.

In British Columbia, Canada, Northern Health launched the Men’s Health Program in 2010 to address the serious health challenges for men living in this rural and remote area including high rates of cancer, occupational deaths, suicides, and deaths attributed to alcohol and tobacco overuse (Northern Health, 2013). The Men’s Health Program consists of activities, men’s groups, education, and community resources, for example, setting-based screenings (glucose, blood pressure, cholesterol), an interactive website (http://men.northernhealth.ca), a men’s “health survival guide” online, social media outreach, and radio campaigns to promote health and prevent illness (Northern Health, 2013). The program was successful in more than 1,000 men being screened for preventable diseases, and community awareness of men’s health has substantially increased (Northern Health, 2013). In order to improve men’s health, it is also important to influence at multiple levels (Malcher, 2009). For example, Australia’s mentoring program “Pathways to Manhood” provides a transition resource for boys to become young men (http://www.pathwaysfoundation.com.au/). Boys participate in the 5-day bush camp work with an older male mentor/father to develop communication and social skills, stronger male relationships, and increased motivation to pursue education and participate in the community (Malcher, 2009). This program disrupts masculine stereotypes and allows boys/adolescents positive ways to express their masculinity. While these community and Web resources are locale specific, nurse practitioners should be aware of and promote similar programs that allow boys and men to challenge cultural stereotypes.

Another community-based Australian program is the Men’s Sheds initiative (Australian Men’s Sheds Association, 2015). Men’s Sheds are locations where men can socialize with other men and participate in a range of activities, for example, woodworking. The aim of this program is to improve men’s psychosocial mental health and wellbeing through acquisition of new skills and social inclusion (Australian Men’s Sheds Association, 2015). Men’s Sheds provide a community and social hub, an equalizing space, a safe and supportive male environment and meaningful male activities (Hansji, Wilson, & Cordier, 2015). There is no pressure to focus on productivity, and in these noncompetitive environments, men build personal and collective strengths, which is a positive approach to men’s health and health promotion (Hansji et al., 2015). Because men and youth of various ages are welcome at Men’s Sheds, intergenerational mentoring can also develop, which positively affects both the mentor and mentee’s psychosocial health (Wilson, Cordier, & Wilson-Whatley, 2013). Drawing from the Australian example, the Men’s Sheds initiative has started to grow and develop across Canada (Men’s Sheds, 2015). The Men’s Sheds program promotes change without amplifying guilt and shame, fosters connections between men’s health and strength, and spreads health promotion through the testimonials of other men. The peer counselling that takes place at Men’s Sheds can be an effective alternative to formal counselling for some men.

In sum, nurse practitioners can direct male patients toward community and web-based resources as a means to foster health promotion. Similarly, such resources can be used to promote traditional PHC services wherein men are provided an array of options and opportunities to do health.

## Conclusion

Many men access EDs rather than PHC wherein illness prevention and health promotion opportunities are lost, and significant morbidities and mortality rates ensue (CIHI, 2005). Nurse practitioners, as health promoters, can champion change in men’s approaches to health, improving their uptake of PHC services and increasing the potential to prevent disease and/or treat emergent illnesses. By engaging men in PHC, leading causes of male mortality—CVD and suicide, which affect older and younger men respectively (Harris & McKenzie, 2006)—might also be quelled. The aforementioned recommendations and strategies while explicitly connected to the work of nurse practitioners—can be reasonably argued as the responsibility of all PHC providers. However, the broad and formative nurse practitioner scope of practice, the pay-for-service model and the public perceptions of nurses as caring and skilled suggests that this PHC provider group can be potent positive influencers for men’s health. After all, nurse practitioners can build trusting relationships with male patients and collaborate toward agreed on health goals. Most important, the focus of nurse practitioners on positive aspects of what men *can do* for their health and on their *strength* and *resilience* to follow through on the agreed on strategies and goals will inevitably advance men’s self-health within and beyond PHC services (Hansji et al., 2015).
